# Model-dependent GD2 upregulation in Ewing sarcoma with the EZH2 inhibitor tazemetostat: Prerequisites for combination with GD2-specific CAR T cells

**DOI:** 10.21203/rs.3.rs-10369352/v1

**Published:** 2026-07-24

**Authors:** Lara Bücker, Bianca Altvater, Jutta Meltzer, Nicole Farwick, Karen E Pollok, Pankita H Pandya, Mohammad R Saadatzadeh, Ramona Meissner, Wolfgang Hartmann, Claudia Rossig, Sareetha Kailayangiri

**Affiliations:** University Children’s Hospital Muenster; University Children’s Hospital Muenster; University Children’s Hospital Muenster; University Children’s Hospital Muenster; Indiana University School of Medicine (IUSM); Indiana University School of Medicine (IUSM); Indiana University Melvin and Bren Simon Comprehensive Cancer Center (IUSCCC), IUSM; Princess Máxima Center; University of Muenster; University Children’s Hospital Muenster; University Children’s Hospital Muenster

## Abstract

The disialoganglioside GD2 is a cell surface marker of neuroblastoma and an attractive target for chimeric antigen receptor (CAR) T-cell therapy. GD2 is also expressed in Ewing sarcoma (EwS), but more heterogeneously and often below the threshold required for CAR-mediated T-cell activation. We previously showed that pharmacological inhibition of the epigenetic regulator Enhancer of Zeste Homolog 2 (EZH2), for example with the approved drug tazemetostat, selectively upregulates GD2 expression in EwS cells in vitro. Here, we evaluated prerequisites for combining EZH2 inhibition with GD2-directed CAR T-cell therapy.

Oral tazemetostat induced GD2 expression in EwS xenografts in vivo, but the effect was model-dependent and not consistently reproducible. In vitro analyses revealed a pronounced impact of EZH2 inhibition on CAR T cell biology. Tazemetostat markedly impaired T cell proliferation and expansion, while enhancing antigen-specific cytolytic activity on a per-cell basis. In contrast, tazemetostat did not measurably affect macrophage polarization under conditions promoting M2-like phenotypes characteristic of the EwS tumor microenvironment.

Together, these findings argue against concomitant administration of tazemetostat with GD2-specific CAR T cells. The variable target induction capacity of tazemetostat in vivo and its recent withdrawal from the market limit the translational potential of the strategy. Systematic in vivo screening of epigenetically active compounds may identify more suitable candidates for robust, clinically relevant modulation of GD2 expression in EwS and other GD2-positive cancers.

## Introduction

Chimeric antigen receptor (CAR)-engineered T cells are potent novel cancer therapeutics in refractory B cell malignancies. In contrast, progress in solid tumors has been substantially slower, largely owing to the limited availability of suitable target antigens. In contrast to B-lineage markers, tumor-selective surface antigens in solid tumors are often heterogeneously expressed and frequently fall below the density required to trigger CAR signaling and T-cell activation[1–3]. Novel concepts that mitigate antigen-negative escape and address intratumoral heterogeneity are needed.

One of the most promising CAR targets in solid tumors is the oncofetal ganglioside GD2, which is expressed at high density on the cell surface of most neuroblastomas[4], as a consequence of the immature neuroectodermal tissue origin of this pediatric cancer. GD2-directed CAR T cell therapy can induce and in some patients maintain remissions in high-risk neuroblastoma, particularly in settings of low tumor burden[5–7], and has shown clinical activity also against GD2-positive tumors of the central nervous system[8]. GD2 expression has been reported in multiple malignancies, including EwS and other mesenchymal cancers of bone and soft tissues[9–13], melanoma[14] and epithelial cancers such as breast cancer[15] and small cell lung cancer[16]. Clinical trials with GD2-directed CAR T cell products have recently started extending the indication beyond neuroblastoma (NCT03373097, NCT03635632, NCT03721068, EUCT20225017252100). But GD2 expression in cancers other than neuroblastoma is less prevalent and often heterogeneous, even within individual tumors and between primary diagnosis and relapse[11, 12]. Consequently, many patients do not meet defined target-expression thresholds to become candidates for GD2 targeting, and GD2-low/GD2-negative subpopulations may evade CAR T cell recognition.

A potential strategy to address this limitation is to combine CAR T cell therapy with agents that selectively increase antigen expression above the activation threshold. In previous work, we found that EwS can be sensitized to GD2-directed CAR T cell therapy via epigenetic modification with inhibitors of Enhancer of Zeste Homolog 2 (EZH2)^[17]^. Tazemetostat is a selective, orally bioavailable small molecule that competitively inhibits EZH2 methyltransferase activity and reduces H3K27 trimethylation to restore expression of genes repressed through PCR2-mediated chromatin silencing. In EwS, tazemetostat reversibly and selectively upregulated surface GD2 in vitro to levels sufficient to trigger robust activation of GD2-specific CAR T cells, via derepression of enzymes required for GD2 biosysnthesis[17]. Independent studies have reported tazemetostat-mediated upregulation in GD2-low subpopulations of neuroblastoma[18] and in other solid cancers, including lung cancer[19] and medulloblastoma[20]. These observations suggest a therapeutic approach in which tazemetostat pretreatment enables and enhances the antitumor efficacy of GD2-directed modalities such as CAR T cells or antibody-based therapies.

Importantly, implementing EZH2 inhibition in combination with adoptive T cell therapy requires careful consideration of EZH2 functions in immune cells. Epigenetic regulation via EZH2 affects lymphocyte development and has been implicated in T cell memory differentiation and activation responses as well as macrophage polarization[21–23]. To inform clinical translation of epigenetic GD2 target modulation, we evaluated the in vivo ability of tazemetostat to upregulate GD2 in EwS xenografts and assessed its impact on GD2-specific CAR T cells and on polarization of myeloid cell populations.

## Results

### Oral tazemetostat induces GD2 in EwS xenografts in a model-dependent manner.

**Figure 1:**
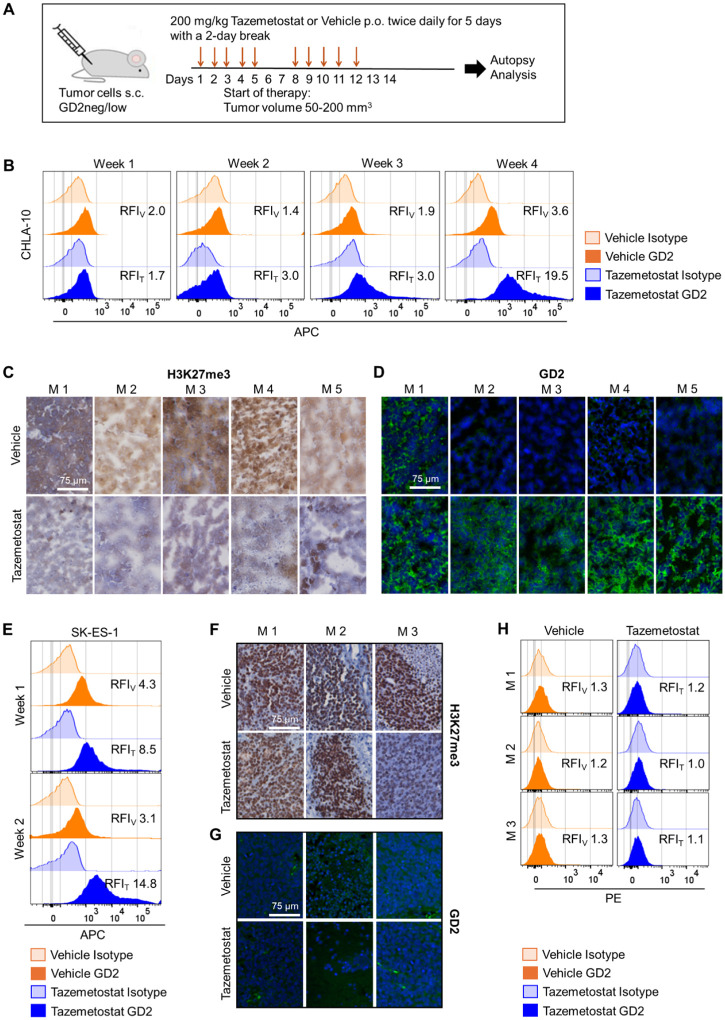
GD2 upregulation in EwS xenografts following EZH2 inhibition in vivo. (A) Experimental design. NSG mice were subcutaneously (s. c.) injected with 5×10^6^ cells of the GD2-negative Ewing sarcoma cell lines CHLA-10 or SK-ES-1 then treated orally with tazemetostat (200 mg/kg BID, 5 days/week) or vehicle for two weeks or until humane endpoint. (B) GD2 expression on CHLA-10 cells cultured in vitro with 1 μM Tazemetostat or vehicle (DMSO) for 28 days, assessed weekly by flow cytometry. (C, D) CHLA-10 xenografts analyzed for H3K27me3 by immunohistochemistry (C) and GD2 expression by immunofluorescence staining (D). (E) GD2 expression on SK-ES-1 cells cultured with 1 μM tazemetostat or vehicle for 14 days, analyzed by flow cytometry. (F, G, H) SK-ES-1 xenografts analyzed for H3K27me3 by immunohistochemistry (F) and for GD2 expression (G) by immunofluorescence staining or flow cytometry (H).

GD2 upregulation in EwS cells via EZH2 inhibition has largely been demonstrated in in vitro culture models. To determine whether pharmacologic EZH2 inhibition upregulates GD2 expression in vivo, NSG mice bearing Ewing sarcoma xenografts were treated with oral tazemetostat (200 mg/kg BID) or vehicle for two weeks or until tumors reached the predefined humane endpoint (Fig. 1A). The primary study endpoints were modulation of H3K27me3 and GD2 expression in harvested tumor tissues.

Our first model relies on the EwS cell line CHLA-10 which lacks baseline GD2 expression (Fig. 1B). Weekly flow cytometric analysis during continuous exposure to tazemetostat demonstrated progressive induction of surface GD2 whereas vehicle-treated cells remained GD2-negative (Fig. 1B). We next examined CHLA-10 tumors harvested from treated mice. Tazemetostat treatment produced a marked reduction in H3K27 trimethylation, confirming on-target pharmacodynamic activity in vivo (Fig. 1C), and was accompanied by robust induction of GD2 expression throughout the CHLA-10 xenografts (Fig. 1D). Vehicle-treated tumors remained GD2-negative/low. Single-agent tazemetostat did not produce an appreciable effect on tumor growth under these treatment conditions (Supplementary Fig. 1).

We next evaluated whether these findings could be reproduced in an independent Ewing sarcoma model. Similar to CHLA-10 cells, SK-ES-1 cells responded to tazemetostat in vitro with increased surface GD2 expression after only two weeks of treatment (Fig. 1E). In contrast, this in vitro response did not translate to the corresponding SK-ES-1 xenografts. Unlike the CHLA-10 model, H3K27 trimethylation was not detectably reduced in SK-ES-1 xenografts following treatment (Fig. 1F), and GD2 expression remained low in tumors from both vehicle- and tazemetostat-treated mice (Fig. 1G, H), suggesting that model-specific biological differences, rather than drug exposure alone, influence the in vivo pharmacodynamic response to EZH2 inhibition.

Collectively, these findings demonstrate that oral tazemetostat can induce GD2 expression in vivo, but that this effect is model dependent and associated with effective inhibition of H3K27 trimethylation.

### Tazemetostat inhibits CAR T cell expansion associated with reduced H3K27 trimethylation.

To inform clinical scheduling of tazemetostat relative to CAR T-cell administration, we investigated how pharmacologic EZH2 inhibition affects the function of GD2-specific CAR T cells. As effector cells, we used GD2IL18CART cells, a product currently in phase 1 clinical investigation (EU CT 20225017252100). These T cells are engineered to express a CAR with 4–1BB costimulation along with NFAT-inducible expression and release of IL-18 cytokine to enhance activity in the TME of solid tumors[24].

**Figure 2:**
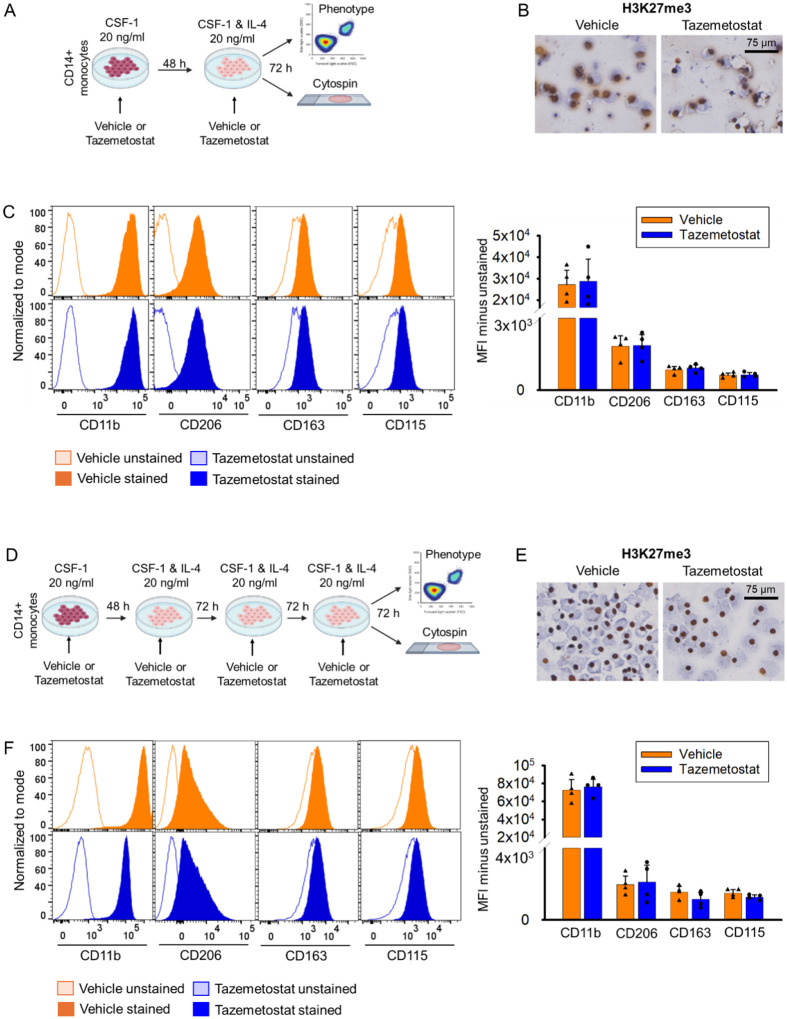
Effects of EZH2 inhibition on macrophage differentiation and polarization. **(A)** Experimental design for differentiation of peripheral blood monocytes into macrophages using M-CSF followed by M-CSF/IL-4 in the presence of 1 μM tazemetostat or DMSO (left). Created partly in BioRender. Altvater, B. (2026) https://BioRender.com/b1guai3. **(B)** H3K27me3 staining is shown for monocytes on day 5 (right). **(C)** Expression of M2-associated macrophage markers (CD11b, CD206, CD163, CD115) after cytokine-driven polarization in the presence of tazemetostat or vehicle in a representative histogram of one donor (left panel) and mean values with standard deviation from four different monocyte donors (right panel). Shown is the median fluorescence intensity (MFI) minus the unstained control. **(D)**Experimental design for long-term macrophage differentiation with M-CSF/IL-4 with tazemetostat or vehicle for 11 days. Created partly in BioRender. Altvater, B. (2026) https://BioRender.com/mkb10hm. **(E)** H3K27me3 staining is shown on day 11. **(F)** Expression of M2-associated macrophage markers after 11-day cultures with tazemetostat or vehicle in a representative histogram of one donor (left panel) and mean values with standard deviation from four different monocyte donors (right panel). After testing for normal distribution, statistical significances were determined with the student’s t-test or Mann-Whitney-U. Only significant differences are marked.

Following non-specific stimulation via CD3/CD28, CAR T cells were treated twice weekly for 3 weeks with tazemetostat (1 μM) or corresponding amounts of DMSO as control, followed by a 2-week washout period (Fig. 2A). EZH2 inhibition by tazemetostat profoundly impaired in vitro expansion of GD2IL18CART cells compared to DMSO control after 3 weeks (median 0.20-fold, range 0.06 to 0.46 vs 3.25-fold, range 1.98 to 4.97) (Fig. 2A). Notably, expansion remained strongly attenuated even after two weeks of withdrawal and despite effective reversal of the effects on H3K27me3 in the absence of tazemetostat (median 0.33-fold, range 0.08 to 0.65 vs 6.42-fold, range 3.03 to 7.00) (Fig. 2A). Western Blot analysis confirmed a marked reduction of H3K27me3, with restoration to baseline levels 2 weeks after withdrawal of tazemetostat (Fig. 2B, left panel). The substantial reduction of CAR T cell expansion under tazemetostat exposure limited the time window for subsequent investigation of CAR T cell phenotypes and function. To obtain sufficient cell numbers for these analyses, the experiment was performed in a shorter, maximum 2-week format. CAR T cells were stimulated and expanded in the presence or absence of tazemetostat as above. Suppression of H3K27me3 after 2 weeks was again confirmed, by immunohistochemistry (Fig. 2B, right panel).

Two weeks of tazemetostat treatment did not affect the proportions of CAR-expressing cells among CD4 + and CD8 + T cells (Fig. 2C). With regard to T cell subset composition, the only statistically significant change was a reduced proportion of T cells with naïve phenotype (TN, CD45RO^−^CD197^+^) among CD4 + T cells (median 0.36%, range 0.12 to 0.78% (tazemetostat) vs. median 1.94%, range 1.28 to 2.61% (vehicle) (Fig. 2D). The inhibitory receptors/exhaustion-associated T cell markers PD-1, TIM-3 and LAG-3 were not significantly affected. Thus, EZH2 inhibition has only limited effects on the phenotypes of the CAR T-cell population maintained in cultures with tazemetostat.

To understand the impact of EZH2 inhibition on the functional response of CAR T cells to antigen, we compared target-specific cytokine production and cytolytic capacity of GD2IL18CART cells after treatment with tazemetostat or DMSO. Secretion of interferon γ (IFN-γ) and tumor necrosis factor α (TNF-α), key mediators of CAR T cell antitumor activity, is further enhanced by antigen-inducible IL-18 secretion in this product[24]. The presence of tazemetostat during 72-hour stimulation of GD2IL18CART cells with the anti-idiotype antibody ganglidiomab (used here to mimic antigen exposure) did not affect secretion of TNF-α and IFN-g, nor of the immune-modulatory cytokines interleukin (IL)-6 and IL-4 or of the TNF superfamily receptor-ligand pair, Fas and FasL (Fig. 2E). Antigen-inducible secretion of IL-18, driven by NFAT promoter activation in our CAR T cell product, also did not vary between the two populations.

We next assessed the effect of tazemetostat on the GD2-specific cytotoxicity of GD2IL18CART cells. Targets were HT1080 fibrosarcoma cells expressing GD2 either at low levels (wild-type) or engineered to express high levels of GD2[24] (Fig. 2F). CAR T cells pretreated with tazemetostat or DMSO for 1–2 weeks were co-cultured with the target cells for 48 hours, and tumor cell death was analyzed by live cell imaging. As expected, cytolytic efficacy was higher against target cells expressing high versus low densities of GD2 (Fig. 2F). Tazemetostat pretreatment did not affect CAR T cell mediated cytolysis against either of the two target cell types (Fig. 2F).

Collectively, the principle effect of EZH2 inhibition on GD2IL18CART cells was a marked impairment of CAR T cell expansion, arguing strongly against concomitant administration of tazemetostat and CAR T cells in a combination strategy.

### Tazemetostat does not affect the phenotypes of in vitro generated macrophages.

The tumor microenvironment (TME) of EwS is frequently enriched in macrophages with immune-inhibitory features which are thought to limit effective tumor immunity[12, 25]. Because macrophage polarization involves epigenetic regulation^[22]^, EZH2 inhibition could, in principle, modulate relevant bystander populations and thereby influence combination strategies.

**Figure 3:**
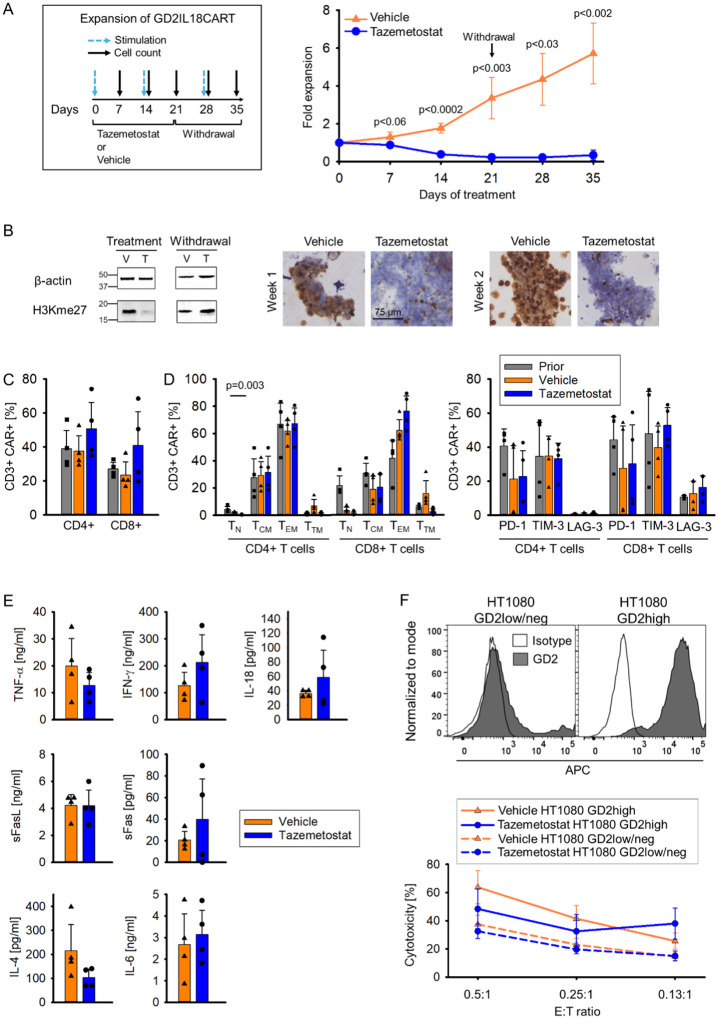
Effects of EZH2 inhibition on CAR T cell expansion and function. **(A)** Experimental design (left panel). GD2IL18CART cells were stimulated with CD3/CD28 antibodies initially and every two weeks. Tazemetostat (1 μM) or DMSO was added for 3 weeks, followed by a 2-week withdrawal period. Expansion of CAR T cells during tazemetostat treatment and after withdrawal, determined by weekly cell counts (right panel) **(B)** H3K27me3 levels in CAR T cells after 4 weeks of treatment and 2 weeks of subsequent withdrawal, respectively, analyzed by Western blot (left panel) and H3K27me3 levels in CAR T cells after one and two weeks treatment analysed by immunohistochemistry (right panel). **(C)** Transduction efficiencies of CD4+ and CD8+ CAR T cells after 2 weeks of treatment with tazemetostat or vehicle. **(D)** Differentiation phenotypes (left panel) and inhibitory marker expression (right panel) of CD4+ and CD8+ CAR T cells after 2 weeks of treatment with tazemetostat or vehicle. T_N_, naïve T cells (CD45RO−/CD197+), T_CM_, central memory T cells (CD45RO+/CD197+), T_EM_, effector memory T cells (CD45RO+/CD197−), T_TM_, terminal memory T cells (CD45RO−/CD197−). **(E)** Cytokine secretion after 2 weeks of treatment with tazemetostat or vehicle and antigen-specific stimulation with ganglidiomab, analyzed by LegendPlex assay. **(F)** Cytotoxicity of CAR T cells pretreated with tazemetostat or DMSO against GD2-positive and GD2 low HT1080 cells, assessed by live-cell imaging over 48 h. Shown as endpoint analysis for the different E:T ratios. Data in panels A and C-F represent mean ± SD of four donors. After testing for normal distribution, statistical significances were determined with the student’s t-test or Mann-Whitney-U, respectively. Only significant differences are marked.

To generate macrophages, CD14 + monocytes were cultured with macrophage colony-stimulating factor (M-CSF) for 48 hours, followed by 3 days of polarization with M-CSF and IL-4 to induce an M2-like phenotype. Tazemetostat (1 μM) or an equivalent concentration of DMSO was present throughout the culture period (Fig. 3A). Five days of tazemetostat exposure did not result in an apparent reduction in H3K27me3 levels in the resulting macrophages (Fig. 3B). The predominant macrophage phenotype under these conditions was characterized by coexpression of CD11b and CD206, with lower expression of CD115 and CD163 (Fig. 3C). Tazemetostat did not affect the proportions of macrophages expressing either of these defining markers (Fig. 3C).

To allow longer exposure to EZH2 inhibition, cultures were extended to 11 days (Fig. 3D). Even after prolonged tazemetostat treatment, no apparent reduction in H3K27me3 levels was observed (Fig. 3E). The same stimulation conditions again generated a macrophage population predominantly coexpressing CD11b and CD206 (Fig. 3F), and tazemetostat did not alter the proportions of cells expressing either marker.

Thus, under the macrophage differentiation and polarization conditions tested here, tazemetostat neither reduced H3K27me3 levels nor measurably altered M2-like polarization.

## Discussion

The capacity of tazemetostat to enhance GD2 surface expression in tumor cells has been independently confirmed in multiple in vitro systems[17–20, 26]. In our hands, translation of this effect to EwS in vivo was model-dependent and not consistently reproducible. Two prior studies reported successful GD2 upregulation in murine tumor xenografts of neuroblastoma, osteosarcoma and medulloblastoma after oral administration of tazemetostat[18, 20]. Mabe et al. administered 350 to 500 mg/kg tazemetostat twice daily (BID) for 28 to 30 days and observed a substantial increase in GD2 expression in metastatic tumors of the GD2-low neuroblastoma cell line SK-N-AS, which translated into significantly increased antitumor activity of both an anti-GD2 antibody and GD2-directed CAR T cells[18]. In heterogeneous tumors derived from the neuroblastoma cell line Kelly, oral tazemetostat treatment eliminated the GD2-low subpopulation[18]. In an orthotopic in vivo model of osteosarcoma, tazemetostat increased GD2 to uniformly high levels in xenografts of MG63.3, which show intermediate/baseline GD2 expression[18]. Ciccone et al. reported increased GD2 density in intracranial orthotopic xenografts of the GD2-positive medulloblastoma cell line Daoy following treatment of the mice with oral tazemetostat (400 mg/kg BID) [20]. H3K27 methylation status of tumors was not reported in these experiments.

With the intention to inform clinical translation in EwS, we used a comparable treatment regimen but selected a lower dose of tazemetostat that approximates human steady state exposures and avoids supratherapeutic levels relative to those typically achieved in patients[27]. We selected two EwS cell lines that were GD2-negative by flow cytometry, defined by relative fluorescence intensities of less than 2, yet inducible by tazemetostat under in vitro conditions. Mice were treated for two weeks with 200 mg/kg tazemetostat BID (5 days on, 2 days off) to maintain tolerability in NSG mice. Under these conditions, the ability of tazemetostat to induce GD2 in vivo varied across models, discouraging immediate clinical translation of a pretreatment strategy in EwS. Besides the lower daily dose of tazemetostat, the weekly 48-hour drug-free interval required for tolerability could, in principle, have contributed to the inconsistencies we observed. We consider this highly unlikely because epigenetic effects of tazemetostat persist beyond drug clearance. Despite the short half-life of tazemetostat of 3 to 4 hours in both mice and humans[28], transcriptional reprogramming is maintained for several weeks after tazemetostat withdrawal[17, 18]. This was documented also in our model by effective H3K27me3 downregulation (Fig. 1C). A longer treatment period (e.g. 21–28 days), demonstrated to achieve H3K27me3 suppression in other tumor xenograft models[29, 30] could potentially increase the likelihood of stable GD2 induction, but was not feasible in our EwS models due to aggressive tumor growth kinetics.

A potential biological explanation for incomplete in vivo target modulation is compensation through EZH1-mediated H3K27 methylation[31], suggesting that dual EZH1 and EZH2 inhibitors such as valemetostat may warrant evaluation. Alternative epigenetic modifiers may also be considered. For example, the deacetylase inhibitor vorinostat has been found to upregulate GD2 in neuroblastoma and enhance anti-GD2 antibody activity[32], although efficacy in EwS remains to be established. Systematic screening of epigenetic modifiers, alone or in rational combinations, may identify clinically applicable agents with greater potency and reproducibility for GD2 induction. Ultimately, the most relevant assessment of clinically approved modifiers will require evaluation in patients. Whole-body GD2 immuno-PET provides a non-invasive approach to quantify GD2 expression across metastatic sites and could support pharmacodynamic monitoring in such studies[33].

Besides robust target upregulation in vivo, combination strategies with T cell–based immunotherapies must account for drug effects on the effector cells themselves and on relevant bystander populations. We observed that tazemetostat significantly reduced the in vitro proliferation and expansion of the CAR T cells in response to a (non-specific) stimulus. This finding is consistent with the established role of EZH2 in cell-cycle progression and early clonal expansion of CD8 + T cells: EZH2 deletion de-represses cell cycle inhibitors and impairs activation-induced T cell expansion[34, 35]. In murine tumor and infection models, EZH2-deficient CD8 + T cells show impaired expansion and reduced functional control upon antigen challenge or rechallenge[36–38]. A similar impairment of proliferative capacity under PRC2 inhibition has also been reported in human T cells[39].

PRC2 activity further contributes to effector terminal differentiation and loss of multipotency[37]. In line with this concept, tazemetostat treatment (200 mg/kg BID) of NSG mice receiving human CD19-specific CAR T cells was associated with an enrichment of transcriptional programs reflecting activated naive/early memory states[40]. Notably, in our study tazemetostat did not compromise antigen-specific killing and even enhanced cytolytic activity on a per-cell basis. Similar findings have been reported previously[35, 39, 41]. One possible explanation is selective outgrowth of cell subsets with derepressed effector-associated genes under continuous EZH2 inhibition and thus an intrinsically stronger cytotoxic machinery [37, 38, 40]. The especially strong killing capacity observed here further reflects the IL-18 armoring of our CAR T-cell product, which augments effector responses upon antigen engagement[24].

Overall, although exposure to tazemetostat may enhance the quality of CAR T cells by shifting toward less differentiated phenotypes with preserved or increased per-cell cytotoxicity, robust in vivo expansion of CAR T cells after antigen engagement remains a key prerequisite for durable antitumor effects. Consequently, the pronounced inhibitory effect of tazemetostat on T cell proliferation argues against concomitant administration with GD2-directed CAR T cells. Instead, to use EZH2 inhibition to upregulate GD2, pretreatment of patients before CAR T cell infusion will be more appropriate to avoid direct suppression of in vivo expansion. An additional consideration for an optimal timing is whether pretreatment might influence the quality of the apheresis product. Notably, manufacturing of CD19 CAR T cells from peripheral blood of patients treated with tazemetostat did not adversely affect functional parameters of the resulting product[40]. Thus, the combination of short half-life[28] and comparatively durable epigenetic effects on cancer cells[17, 18] may create a practical window in which CAR T cells could be administered after drug wash-out while tumor GD2 density remains elevated.

In contrast to T cells, tazemetostat did not produce an apparent reduction in H3K27me3 staining or alter macrophage polarization under either of the M2-polarizing conditions examined. Published data on the effects of EZH2 inhibition in macrophages are heterogeneous and highly context dependent. Whereas two studies reported enhanced M1-like polarization in glioblastoma and colorectal carcinoma models, respectively[42, 43], others observed promotion of M2 polarization in different disease settings[44, 45]. Species-specific differences may further contribute to these discrepancies. In human monocytes and monocyte-derived macrophages, exposure to 10 μM tazemetostat for 24–48 hours reduced H3K27me3 levels and altered inflammatory gene expression[46]. A key difference in our study is that we used a 10-fold lower concentration of tazemetostat (1 μM), reflecting clinically attainable serum levels at standard dosing[27], which may explain the absence of detectable effects on H3K27me3 and macrophage phenotype. Given the well-known plasticity of in vitro macrophage cultures, analysis of tumor biopsies from patients receiving tazemetostat or other EZH2 inhibitors may provide a more informative assessment of the effects of EZH2 inhibitors on human myeloid populations in vivo.

In summary, under pharmacologically relevant dosing designed to approximate clinical exposure, oral tazemetostat produced inconsistent GD2 induction in EwS xenograft models, discouraging immediate clinical translation of a pretreatment strategy for GD2-directed therapies. Strong inhibitory effects on activation-induced expansion of (CAR) T cells further argue against concomitant administration. Moreover, the clinicial use of tazemetostat has become unfeasible in the meantime: Following FDA accelerated approvals in 2020 for epithelioid sarcoma[47] and for relapsed/refractory follicular lymphoma[48] and phase 2 investigation in pediatric cancer cancers[49], tazemetostat was voluntarily withdrawn from the market in 2026 following emerging safety data indicating an increased risk of secondary hematologic malignancies. Identification of more effective and safe epigenetic approaches for GD2 modulation and systematic characterization of their effects on tumor cells and immune effector populations could still lead to a viable route to address antigen heterogeneity in EwS and other GD2-positive cancers.

## MATERIALS AND METHODS

### General laboratory operation.

The assays were performed by experienced individuals throughout the course of the study, using established laboratory protocols covering the processing, freezing, storage, and thawing of cells as well as the staining procedure, data acquisition, and gating strategy. All experiments were performed and reported in accordance with the relevant guidelines and regulations. Raw data can be provided upon request.

### Cell culture.

The Ewing cell line SK-ES-1 (RRID:CVCL_0627) was purchased from DSMZ (Braunschweig, Germany). CHLA-10 cells (RRID:CVCL_6583) were obtained directly from the COG by Dr. Jake Zucker (Karen Pollok group) or from the Health Sciences center at Texas Tech University (Rossig group). DNA fingerprinting analysis using a nine-marker short-tandem repeat (STR) analysis (IDEXX BioResearch, Columbia, MO, USA) was used to authenticate for their identity, as previously described[50, 51]. CHLA-10 cells were cultured in DMEM supplemented with 10% FBS and 1% HEPES and SK-ES-1 was cultured in RPMI supplemented with 10% FBS and 1% Glutamine in collagen-coated flasks at 37°C in a humidified atmosphere containing 5% CO2. A GD2-positive variant of the fibrosarcoma cell line HT1080 (RRID:CVCL_0317) was generated as previously reported[52] and transduced with Incucyte^®^ Nuclight Orange Lentivirus (Sartorius, Göttingen, Germany). The Lenti-X 293T cell line was purchased from TaKaRa (632180). Tumor cells were cultured in rat tail collagen (Z-17C03-C Cell Concepts, Germany) coated 25- or 75-cm^2^ tissue culture flasks in RPMI 1640 medium (21875–034 Gibco, Germany), supplemented with 10% heat-inactivated fetal calf serum (FCS; P30–3031 PAN-Biotech, Germany) and 2 mM L-glutamine (G7513 Sigma-Aldrich, Germany), and maintained at 37°C and 5% CO_2_. Lenti-X 293T cells were cultured in IMDM (21980–032 Gibco, Germany) with 10% FCS and 1% penicillin (100 IU/ml)/streptomycin (100 mg/ml) (P4333 Sigma-Aldrich, Germany). The identity of the tumor cell lines was confirmed by short tandem repeat profiling. All cells were mycoplasma negative. T cells and monocytes were isolated by density gradient centrifugation with BioColl separation solution (BS.L6115 BioSell, Germany) from leucocyte-reduction system chambers of healthy donors provided by the blood bank from University Hospital Muenster upon approval by the University of Münster Ethical Board (1IXRös1).

### In vivo experiments.

All mouse experiments in this study were in accordance with the ARRIVE guidelines 2.0. The primary endpoints of both studies were modulation of H3K27me3 and GD2 expression in harvested tumor tissues.

For the **CHLA-10 xenograft study**, NOD.Cg-Prkdc Scid Il2rgtm1Wjl/SzJ (NSG) mice (8–10 weeks old) were obtained from the on-site breeding colony maintained by the In Vivo Therapeutics Core (IVT Core) at the Indiana University Melvin and Bren Simon Comprehensive Cancer Center. All animal procedures were approved by the Indiana University Institutional Animal Care and Use Committee (IACUC Protocols 22028 and 25041) and were conducted in accordance with institutional guidelines for the care and use of laboratory animals. Mice were housed under specific pathogen-free conditions with ad libitum access to Teklad Laboratory Diet (TD2018SX, Inotiv, Indianapolis, IN, USA) and reverse osmosis (RO) water on a 12-hour light/dark cycle at 22–24°C with standard environmental enrichment. Animals were acclimated for at least 7 days before tumor implantation. Mice were injected subcutaneously in the right flank with 5 × 10^6^ CHLA-10 cells suspended in 200 microliters of Matrigel^®^ Matrix Basement membrane (cat. # 354234, CORNING). Tumor growth was monitored three times weekly using electronic calipers interfaced with Study Director software (Studylog Systems, Inc.). Mice with tumor volumes between 50–200 mm^3^ were eligible for enrollment and were block randomized to treatment groups using Study Director software to achieve balanced baseline tumor volumes. Four mice were excluded prior to treatment allocation because tumor volumes did not meet the predefined inclusion criterion. The study was designed as an exploratory pharmacodynamic study to obtain tumor tissue for molecular analyses rather than to evaluate treatment efficacy. Accordingly, no a priori power calculation was performed. Sample sizes (n = 5 mice/group) were selected based on prior experience to provide sufficient tumor tissue for the planned pharmacodynamic analyses. Mice received either vehicle (0.5% sodium carboxymethylcellulose and 0.1% Tween-80 in water; n = 5) or tazemetostat (200 mg/kg; HY-13803, MedChemExpress; n = 5) by oral gavage twice daily (6-hour interval) for three treatment cycles, each consisting of five consecutive treatment days with two-days off. Tumor growth and animal health were monitored throughout the study in accordance with the approved IACUC protocol. Mice were euthanized when tumors reached the predefined humane endpoint of approximately 2000 mm^3^. Body weights were maintained throughout the study. Tumors were harvested immediately for downstream molecular analyses. All tumors included in the study were collected while animals remained under therapeutic drug exposure. Personnel in the IVT Core, who were independent of the Pollok laboratory, were aware of treatment allocation during drug administration and tumor monitoring.

The **SK-ES-1 study** was approved by the State Agency for Nature, Environment and Consumer Protection (LAVE, Recklinghausen, Az. 2024 − 127). Female NSG mice aged 7–10 weeks were ordered from Charles River (Sulzfeld, Germany). Before starting the experiment, the mice were familiarized with their new surroundings in the central animal experimental facility Muenster (ZTE) for two weeks. They were housed in individually ventilated 2L (long) cages (IVC, Charles River) with a maximum of five animals per cage. They had free access to sterile food and water and the temperature was kept constant at 21°C. Sample size (n = 3) were chosen by using the Resource Equation method described by Festing et al. Mice were randomized per cage and no blinding was performed. SK-ES-1 tumor cells (5×10^6^) were resuspended in RPMI and subcutaneously injected into the right flank to allow for local tumor growth. The mice were anesthetized with Isoflurane prior to the procedure. Inhalation anesthesia is induced with 4% (v/v) isoflurane in oxygen using an induction chamber. Anesthesia is maintained with 1.5–2.5% (v/v) isoflurane in oxygen, delivered via a face mask. Tumor cell engraftment and tumor growth were monitored at regular intervals. Treatment with tazemetostat (200 mg/kg BID, HY-13803 MedChemExpress) dissolved in vehicle solution (0.5% sodium carboxymethylcellulose (419273 Sigma-Aldrich, Germany) and 0.1% Tween-80) or equivalent amount of vehicle solution alone was administered twice daily (6 hour interval) for two weeks by oral gavage when tumor volumes reached 100–200 mm^3^. Tumor sizes were assessed with a caliper. Body weights were maintained throughout the study. Five mice were sacrificed two days after the last tazemetostat administration. One mouse was euthanized earlier because the predefined humane endpoint criteria were met. For euthanization, mice are first anesthetized with isoflurane, then cervical dislocation is performed by displacing the skull and cervical vertebrae relative to each other. Tumors were harvested and used for subsequent analysis.

### GD2 staining by flow cytometry.

Tumor cells (50,000 each) from in vitro cultures were stained with PE-conjugated monoclonal antibody against GD2 (clone 14.G2a, RRID:AB_2561885, 357304 Biolegend, Germany) or corresponding isotype IgG2a-PE (clone MOPC-173, RRID:AB_326460, 400212 Biolegend, Germany). Samples were measured with FACS Diva 8.0 using a FACS Celesta flow cytometer (RRID:SCR_019597, BD Biosciences, Germany) and analyzed using FlowJo version 10 (RRID:SCR_008520, FlowJo, USA). Relative fluorescence intensities were calculated by dividing median fluorescence intensities of GD2-stained cells by those obtained with isotype antibody. To analyze tumor cells from murine xenografts, the tumor tissues were smashed and filtered through a cell strainer (100 μm) to obtain a single cell suspension, then incubated in erythrocyte lysis buffer (79217 Qiagen, Germany) for 7 min. After a washing step, the cells were stained with the following antibodies: CD45-PerCp (clone 30-F11, RRID:AB_893339, 103130 Biolegend, Germany), GD2-PE (clone 14.G2a, RRID:AB_2561885, 357304 Biolegend, Germany), CD99-FITC (clone 3B2/TA8, RRID:AB_2616972, 371304 Biolegend, Germany) and the corresponding isotypes IgG2a-PE (clone MOPC-173, RRID:AB_326460, 400212 Biolegend, Germany) and IgG2a-FITC (clone MOPC-173, RRID:AB_326458, 400210 Biolegend, Germany), then analyzed as above with a gate on CD99 positive cells.

## GD2 immunofluorescence staining of tumor tissue sections

Formalin-fixed paraffine-embedded (FFPE) material was assessed for GD2 expression as previously described in detail[53]. In brief, tissue sections were deparaffinized with xylene and rehydrated in decreasing concentrations of ethanol. Antigen unmasking was performed in a steam cooker. Endogenous peroxidases were blocked with 3% H_2_O_2_ diluted in PBS, followed by blocking with undiluted normal goat serum (927503 Biolegend, Germany) for 1 h at RT. Slides were incubated overnight at 4°C with the unconjugated primary anti-GD2 antibody (Clone 14.G2a, RRID:AB_395336, 554272 BD Pharmingen) diluted 1:50 in PBS supplemented with 10% normal goat serum. Slides were then incubated with HRP-conjugated secondary antibody (Polymer HRP Ms + Rb, ARH1001EA Akoya Biosciences) for 10 min at RT followed by staining with Opal fluorophore 520 (1:100, FP1487001KT Akoya Biosciences) for 10 min at RT. All slides were stained with DAPI (GTX16206 Biozol, Germany) and enclosed with Prolong Diamond Antifade solution (P36970 Invitrogen).

To detect GD2 on cryostat-frozen tumor tissues, sections of 4–5 μM were fixed in 2% paraformaldehyde for 10 min, then permeabilized for 3 hrs in phosphate-buffered saline (PBS) containing 0.075% Tween and 1% bovine serum albumin (BSA). After washing, slides were rinsed in PBS supplemented with 1% BSA and then incubated overnight at 4°C with the unconjugated primary anti-GD2 (Clone 14.G2a, RRID:AB_395336, 554272 BD Pharmingen) antibody diluted 1:50 in PBS containing 0.01% Tween. Five 5-min washes in PBS were conducted after each incubation. The slides were incubated with a polyconal FITC-conjugated goat anti mouse antibody (BD Pharmingen) diluted 1:1000 in PBS for 30 min at room temperature (RT). The slides were counterstained with DAPI (BioCat) and enclosed with Prolong Diamond Antifade (Thermo Fisher) solution. Imaging was performed with the Vectra^®^ 3.0 system. One area of the slides was randomly selected and analyzed using InForm Analysis software (RRID:SCR_019155).

### Immunohistochemistry (IHC) analysis of H3K27me3.

FFPE material of tumor tissues was deparaffinized in xylene and dehydrated in a decreasing series of alcohols (99%, 96%, 70%). Antigen unmasking was performed in citrate buffer (pH 6) in the microwave until boiling and then for 20 min at 95–98°C. After cooling for 20 min at RT, peroxidase block was performed in 3% H_2_O_2_ in distilled water for 10 min. Protein blocking was performed with Tris-Buffered Saline with Tween 20 (TBST) and 5% normal goat serum (927503 Biolegend, Germany) for 1 h at RT in a humidified chamber. The slides were incubated overnight at 4°C in primary antibody Tri-Methyl-Histone H3 (Lys27) (RRID:AB_2616029, 9733 Cell Signaling, Germany) diluted 1:400 in antibody diluent. Incubation with the secondary biotinylated antibody goat-anti-rabbit (RRID:AB_2533969, 65–6140 Thermo Fisher Scientific, Germany) diluted 1:100 in antibody diluent was performed at RT for at least 30 min in a humidified chamber. Subsequently, incubation with ABC reagent (Vectastain^®^ Elite^®^ ABC Kit, PK-6100 Vector Laboratories, USA) was performed for 20 min at 37°C. After washing with TBS, the sections were stained with the DAKO Substrate Chromogen Kit (K3468 DAKO) according to the manufacturer's instructions until a color change was visible. Finally, counterstaining with haemalaun (1.09249 sigma Aldrich, Germany) was performed, followed by dehydration in a series of increasing alcohol concentrations (70%, 96%, 99%) and covering with mounting medium (Vitro-Clud, 04 − 0001 Langenbrinck, Germany). Imaging was performed as described above.

Cryo-slides of xenograft tissue or cytospins of immune cells were first incubated for 30 min at RT, then for 10 min in acetone (5025.2 Roth, Germany) at −20°C. After washing in PBS, the endogenous peroxidases were blocked with 0.3% H_2_O_2_ in PBS for 10 min followed by protein blocking and H3K27me3 staining as described above.

### CAR T cell generation.

Particles of the SIN lentiviral vector GD2CAR-IL18 were generated as described[24, 52]. Primary human T cells were isolated from peripheral blood mononuclear cells (PBMC) by Magnetic activated cell sorting (MACS^®^ Milteny Biotec, Germany) and stimulated using Immunocult^™^ Human CD3/CD28 T cell Activator (25 μl/ml, 10991 StemCell, Germany) in TexMACS medium containing 3% human AB serum, 1% P/S, human IL-7 (500 IU/mL) and human IL-15 (84 IU/mL). After 48 h, lentiviral particles were added together with Synperonic^®^ F 108 (500 μg/ml, 07579–125G-F Sigma Aldrich, Germany). Cells were washed after 24 h and resuspended in complete TexMACS medium without human serum. After washing/splitting every two days with fresh TexMACS media containing human IL-7 and human IL-15, the final CAR T-cell product was harvested at day 14 after T-cell isolation, analyzed for transduction efficiency by staining with anti-idiotype antibody as described[24], then used for functional analyses.

### CAR T cell expansion.

At day 14 of CAR T cell cultures, T cells were adjusted to a CAR transduction efficiency of 28–30%, then 15–20×10^6^ T cells per flask were added to 75 cm^2^ suspension cell culture flasks containing Panserin (1 ml/1×10^6^ cells; P04–71413 PAN-Biotech, Germany), human IL-2 (50 IU/mL; 11340023 ImmunoTools) and either 1 μM tazemetostat (16174 Cayman Chemical Company, Germany) dissolved in DMSO (8417 sigma Aldrich, Germany) or an equivalent amount of DMSO. At the start of cultures and then every 2 weeks, the cells were non-specifically re-stimulated with Immunocult^™^ Human CD3/CD28 T cell Activator (25 μl/ml). Medium was changed twice weekly. After three weeks of culture in the presence of tazemetostat, the agent was withdrawn for 2 further weeks. Cells were counted weekly by trypan-blue exclusion, and fold expansion was calculated.

### Western Blot analysis of H3K27me3.

Tumor cells were homogenized in lysis buffer, shortly fractured in liquid nitrogen, thawed on ice, and clarified by spinning. Samples (20 μg) were separated by electrophoresis on an SDS 15% polyacrylamide gel and then electroblotted onto a nitrocellulose membrane (1620112 Bio-Rad, Germany). Blocking was done for 1 h, followed by incubation with anti-H3K27me3 antibody (Clone mAbcam 6002, RRID:AB_305237, ab6002 Abcam, Germany) diluted 1:1,000 in TBST containing 5% BSA overnight at 4°C. Membrane was then incubated with horseradish peroxidase (HRP)-linked anti-mouse immunoglobulin G (IgG) whole Ab (GENA931–1ML Cytiva, Germany) at 1:2,000 in TBST 5% BSA for 1 h at RT, followed by treatment with enhanced chemiluminescence reagent (ECL, RPN2106 Cytiva) and directly analyzed by the Imager ECL Chemostar+ (INTAS Science Imaging, Germany). Equal protein loading was determined by incubation with 7 mL Restore Plus stripping buffer (46430 Thermo Scientific, USA) for 15 min at RT. Blocking was done with TBST buffer with 5% nonfat dry milk (T145.2 Roth, Germany) for 1 h and detection with a β-actin-specific antibody (49675 Cell Signaling Technology, Germany) diluted 1:5,000 in TBST with 5% BSA overnight at 4°C. After washing, the membrane was incubated with HRP-linked anti-rabbit antibody (GENA934–1ML Cytiva) 1:2,000 in TBST 5% milk for 1 h, followed by detection as described above. Uncropped, full-length original gel images are shown in Supplementary Fig. 2.

### Cytokine secretion.

Flat-bottom 96-well plates were coated with the anti-idiotype antibody ganglidiomab for antigen-specific CAR T cell activation[54] (0.5 μg/ml) and incubated at 4°C. After 4 h of coating, the wells were washed with RPMI supplemented with 10% FCS, then 25,000 CAR T cells per well were seeded in Panserin (50 μl/well) in triplicates and incubated at 37°C. After 72 h, cell culture supernatants were carefully collected, pooled, centrifuged and frozen at −80°C until analysis. Cytokine concentrations (TNFα, IFNγ, TGFβ, soluble Fas, soluble FasL, IL-6, IL-4 and IL-18) were quantified by a custom Legendplex Multi-Analyte Flow Assay Kit (Biolegend, Germany). The assay was performed according to the manufacturer's instructions and data analyzed using Biolegend's Qognit Software Version 2025–05-01.

### Cytotoxicity Assay.

Target-specific cytolysis of tumor cells was quantified using the Incucyte^®^ SX5 Live Cell Analysis System (RRID:SCR_026298, Sartorius, Germany). Nuclight orange-positive GD2-positive and GD2-negative/low HT1080 cells (5,000 cells/well) were cocultured with GD2IL18CART cells (adjusted for CAR-expressing cells) at three different effector to target (E:T) ratios (0.5:1, 0.25:1, 0.13:1) in Panserin with low dose IL-2 (50 IU/ml) for 48 h. One row containing tumor cells alone served as control. Incucyte^®^ Cytotox NIR Dye (1:1000, 4846 Sartorius, Germany) was added to allow monitoring of viability, which was done at 4 h intervals using a 10x objective lens with phase contrast and Orange/NIR Optical Module. Tumor cell lysis was analyzed by creating a detection mask, using Nuclight orange and NIR Dye as combined markers for dead tumor cells. The percentage of dead cells per well was calculated by dividing the number of dead cells by the total number of cells and multiplying by 100 to determine the percentage. The percentage of dead cells at 48 h was used for the endpoint analysis.

### CAR T cell phenotyping by flow cytometry.

Prior to staining, all cells were washed with wash buffer (PBS with 0.2% BSA (A7030 sigma Aldrich, Germany) and 0.1% sodium azide (71289 sigma Aldrich, Germany)). Up to 1×10^6^ cells were stained for 15 min at RT in the dark with the following antibodies: CD3-APC-Fire 750 (clone SK7, RRID:AB_2572114, 344840 Biolegend, Germany), CD4-AlexaFluor 700 (clone SK3, RRID:AB_2563150, 344622 Biolegend, Germany), CD8-PerCP (clone RPA-T8, RRID:AB_893421, 301030 Biolegend, Germany) and CF488-conjugated anti-GD2 anti-idiotype antibody ganglidiomab (100 ng, Lode et al. 2013). After a wash step with washing buffer, the cells were divided into panels and stained for 10 min with additional antibodies: antibodies CD45RO-BV605 (clone UCHL1 (RUO), RRID:AB_2744411, 562791 BD Horizon, Germany) and CD197-PE (clone G043H7, RRID:AB_10913813, 353218 Biolegend, Germany) were used to analyze the effector phenotype. CD279 (PD-1)-APC (Clone Eh12.2H7, RRID:AB_940475, 329908 Biolegend, Germany), CD366 (TIM-3)-PE (clone F38–2E2, RRID:AB_2572604, 12–3109-42 eBioscience) and CD223 (LAG-3)-APC (Clone 3DS223H, RRID:AB_2573185, 17–2239-42 eBioscience) were used for the analysis of inhibitory markers. After a subsequent washing step, the cells were fixed in 1% Paraformaldehyde (PFA, P6148 sigma Aldrich, Germany) prior to analysis. Gating is shown in the Supplementary Fig. 3A and B.

### In vitro differentiation of macrophages.

CD14 + cells were isolated from human PBMCs by Magnetic-activated cell sorting (MACS^®^ Milteny Biotec, Germany) with Pan Monocyte Isolation Kit (130–096-537 Milteny Biotec, Germany) according to the manufacturer′s instructions. Samples of 1.4–1.8×10^6^ cells per well were seeded on a Costar^®^ 6-well Clear Flat Bottom Ultra-Low Attachment Plate (3471 Corning, Germany) in Macrophage Base Medium XF (C-28057 PromoCell GmbH, Germany) (Day − 2). After 4 h, the medium was replaced with Panserin supplemented with 20 ng/ml M-CSF (11343115 Immuntools, Germany).

For further cytokine stimulation, 48 h later (day 0) the medium was replaced by Panserin supplemented with 20 ng/ml M-CSF and 20 ng/ml IL-4 (1130045 Immunotools) alone, together with 1 μM tazemetostat dissolved in DMSO or DMSO alone. For 5-day cultures, fresh tazemetostat or DMSO was added on day 3. For 11-day cultures, the medium was completely replaced with fresh cytokines and tazemetostat or DMSO on days 3 and 6. On day 5 or day 11, respectively, macrophage phenotypes were analyzed by flow cytometry, and approximately 0.4×10^6^ cells were fixed by a cytospin (Cytology centrifuge Cellspin^®^ I, Tharmac), followed by H3K27me3 IHC staining to verify EZH2 inhibition.

### Macrophage phenotyping by flow cytometry.

Macrophages were detached from the plates for 20 min at 4°C and washed with wash buffer. All samples were first incubated for 5 min with True-Stain Monocyte Blocker^™^ (426103 Biolegend, Germany) to block non-specific binding and then for 5 min with Human TruStain FcXTM (Fc Receptor Blocking Solution, 422302 Biolegend, Germany) to block unwanted staining involving FcR. Cells were then incubated with the following antibodies for 15 min at RT in the dark: CD115 (CSF1R)-PE (Clone 9–4D2–1E4, RRID:AB_2276697, 347303 Biolegend, Germany), CD206-APC-Fire-750 (Clone 15 − 2, RRID:AB_2650957, 321134 Biolegend, Germany), CD163-Vio Bright B515 (Clone REA812 GHI/61.1, RRID:AB_2904768, 130–112-127 Miltenyi, Germany) and CD11b-APC (Clone ICRF44, RRID:AB_398456550019, BD Pharmingen, Germany). A fluorescence minus one control was implemented for each antibody as a gating control and gating is shown in the Supplementary Fig. 3C.

### Graph design and statistics.

The data was visualized as graphs using SigmaPlot 13 and is presented as mean and standard deviation (SD). Illustrations were created using BioRender. All statistical tests were performed using SigmaPlot software version 13 (RRID: SCR_003210) and normal distribution was automatically tested by the software. The student’s t-test was used to test for significance of samples with parametric distribution and the Mann-Whitney-U test was used to compare means of data sets with non-parametric distribution.

## Supplementary Files

This is a list of supplementary files associated with this preprint. Click to download.

- SupplementaryData.pdf

## Data Availability

All data generated or analyzed during this study are available within this paper.
